# Reversal of Age-Related Learning Deficiency by the Vertebrate PACAP and IGF-1 in a Novel Invertebrate Model of Aging: The Pond Snail (*Lymnaea stagnalis*)

**DOI:** 10.1093/gerona/glu068

**Published:** 2014-05-20

**Authors:** Zsolt Pirger, Souvik Naskar, Zita László, György Kemenes, Dóra Reglődi, Ildikó Kemenes

**Affiliations:** ^1^Balaton Limnological Institute, Centre for Ecological Research, Hungarian Academy of Sciences, Tihany, Hungary.; ^2^Sussex Neuroscience, School of Life Sciences, University of Sussex, Brighton.; ^3^Department of Anatomy MTA-PTE, “Momentum” PACAP Team, University of Pécs, Hungary.

**Keywords:** PACAP, Learning, Memory.

## Abstract

With the increase of life span, nonpathological age-related memory decline is affecting an increasing number of people. However, there is evidence that age-associated memory impairment only suspends, rather than irreversibly extinguishes, the intrinsic capacity of the aging nervous system for plasticity (1). Here, using a molluscan model system, we show that the age-related decline in memory performance can be reversed by administration of the pituitary adenylate cyclase activating polypeptide (PACAP). Our earlier findings showed that a homolog of the vertebrate PACAP38 and its receptors exist in the pond snail (*Lymnaea stagnalis*) brain (2), and it is both necessary and instructive for memory formation after reward conditioning in young animals (3). Here we show that exogenous PACAP38 boosts memory formation in aged *Lymnaea*, where endogenous PACAP38 levels are low in the brain. Treatment with insulin-like growth factor-1, which in vertebrates was shown to transactivate PACAP type I (PAC1) receptors (4) also boosts memory formation in aged pond snails. Due to the evolutionarily conserved nature of these polypeptides and their established role in memory and synaptic plasticity, there is a very high probability that they could also act as “memory rejuvenating” agents in humans.

It is well known that aging affects memory, but only recently has a likely major molecular cause of age-related memory loss been discovered. In a gene expression study in the human dentate gyrus, 17 genes were identified that showed age-related changes (5). The most significant change was an age-related decline in RbAp48, a histone-binding protein that modifies histone acetylation. Inhibition of RbAp48 in young mice caused hippocampus-dependent memory deficits similar to those associated with aging. On the other hand, upregulation of RbAp48 in the dentate gyrus of aged wild-type mice ameliorated age-related hippocampus-based memory loss (5). RbAp48 is known to interact with a complex of CREB binding protein (CBP, a histone acetyl transferase) and phosphorylated CREB1 (pCREB1) (6) and thus it was hypothesized that enhancement of the PKA-CREB1-CBP pathway and the RbAp48 protein could be a target for therapeutic interventions targeting age-related memory loss (5).

The PKA-CREB1-CBP pathway can be enhanced by activating adenylate cyclase, for example, by the application of pituitary adenylate cyclase activating polypeptide (PACAP). Since its discovery in 1989 as a hypothalamic-releasing factor, PACAP has been described to have widespread distribution including expression in the pituitary, gonads, placenta, intestinal tract, adrenal gland, and central and peripheral nervous systems (7). PACAP38 has been found to affect both synaptic plasticity and memory processes in a number of previous studies in vertebrates (8–13) and in invertebrates as well (3). It was shown that PACAP38 facilitates memory retrieval processes in the extinction of the active avoidance reflex in rats (13) and acts as an enhancer of mammalian mnemonic processes even at very low dosages (12). PACAP38 acts through specific PAC1, VPAC1 and VPAC2 receptors, but PAC1 has the largest affinity to the polypeptide (14).

The bioactive forms of PACAP and its receptors have been remarkably well conserved during evolution (14). There is little difference (only three or four amino acids) between the amino acid sequences of PACAP38 in vertebrates and invertebrates (15). This close relatedness provides a good theoretical base to study the role of PACAP38 in mechanisms of memory formation in simpler neuronal systems. It has already been described that PACAP38 and its receptors are present in the pond snail *Lymnaea stagnalis* (2), a well-established invertebrate model organism to study evolutionary conserved molecular mechanisms of associative learning and memory (16,17). There is also evidence that this polypeptide is both necessary and instructive for long-term memory (LTM) formation after food-reward classical conditioning in young *Lymnaea* (3).

A number of previous studies have demonstrated age-related impairment of associative memory in *Lymnaea* (18,19). Other studies characterized age-related changes in key identified neurons of the feeding and respiratory network, the circuits used for classical conditioning and operant conditioning, respectively (1,20–23). Two of these studies (1,22) identified specific electrical changes in identified neurons as the likely underlying cause of age-related memory loss.

A detailed knowledge of the conserved molecular cascades (eg, cAMP-PKA-CREB1-CBP) underlying behavioral LTM in *Lymnaea* (17), together with the already existing information on the cellular and molecular mechanisms of age-related memory loss in this species (1,22) provided us with an ideal model system in which to test the hypothesis that PACAP could reverse memory loss in older age. Here we used a combination of molecular and behavioral methods to measure endogenous PACAP expression levels in the “learning ganglia” of young and aged snails and to test the effect of PACAP on memory in older animals. IGF-I supplementation in rodents has been reported to ameliorate hippocampal-dependent cognitive deficits associated with normal aging (24), but its effect on LTMafter classical conditioning has not been investigated. Based on the finding that insulin-like growth factor-1 (IGF-1) transactivates PACAP type I (PAC1) receptors and 86% of the downstream protein targets are shared between the PACAP- and IGF-1-activated pathways (4), we also tested the effect of IGF-1 on memory formation in aged animals.

## Materials and Methods

### Experimental Animals

Pond snails (*L. stagnalis*) were bred at the University of Sussex. Animals of different ages were kept in separate large holding tanks filled with Cu^2+^-free water (18–20°C). Snails were kept under 12-hour light–dark cycles and fed ad libitum with lettuce and a vegetable-based fish food (TETRA Werke). They were food deprived for 2 days before the beginning of the experiments.

Although snails survive over a year in our breeding facility, two age categories, 3 and 8 months old, were selected based on the age categorization by Hermann and coworkers (19), to compare learning performance in young and older animals.

### Training Procedure

A single-trial appetitive classical conditioning protocol was used (3,25,26) to train young and older animals (aged 3 and 8 months, respectively). Before training, the animals were left in the experimental dish (14-cm diameter Petri dish) for 10 minutes to acclimatize. Then the conditioned stimulus (CS), amyl acetate (0.004% final concentration), was introduced, followed by the unconditional stimulus, sucrose (0.67% final concentration), 15 seconds later. The CS and unconditional stimulus pairing lasted for 2 minutes, and the animals were rinsed and placed back to their original holding tanks. Memory tests were performed 1 hour and 24 hours after the training. The 1 hour represents intermediate-term memory (ITM), whereas the 24-hour time point refers to LTM in *Lymnaea* (27). For testing, individual snails were taken from their home tanks using a blind procedure and placed in Petri dishes. After a 10-minute acclimatization period, rasps were counted for 2 minutes (ie, spontaneous rasping in the absence of the CS). Five milliliters of the CS was then applied to the dish, and rasps were counted for further 2 minutes (ie, rasping in the presence of the CS). The feeding response to the CS was defined as the number of rasps in the presence of CS minus the number of spontaneous rasps.

### Treatment of Animals With a PACAP Receptor Antagonist or PACAP38

Experimental animals were injected with 100 μL of PACAP38 (1.0 μM final concentration) diluted in saline, saline, or antagonist, PACAP6-38 (2.5 μM final concentration) 60 minutes before training.

### Treatment of Animals With IGF-1

Aged animals were injected with 100 μL of IGF-1 (1.3 μM final concentration) diluted in saline or IGF-1 combined with M65, a specific antagonist of PAC1 receptors (2 μM final concentration) 60 minutes before training.

### Western Blot Assays

#### Protein sample preparation.

*Lymnaea* protein samples for western blot were prepared from buccal and cerebral ganglia dissected from young and aged snails and collected on dry ice. Proteins from ganglia were solubilized using ice-cold Tris homogenization lysis buffer (20mM Tris pH 7.5, 1mM ethylene diamine tetra-acetic acid, 5mM dithiothreitol, 10% Glycerol, 1% Protease inhibitor, and 1% Triton X-100) and incubated for 30 minutes on rocker at 4°C. Later, samples were centrifuged at 10,000*g*, and resultant supernatants were collected and purified using chilled acetone. Precipitated proteins were centrifuged and air dried at room temperature before solubilization in Tris buffer (125mM Tris–HCl, pH 6.8).

#### Sodium dodecyl sulfate–polyacrylamide gel electrophoresis and immunoassays.

Purified and solubilized protein samples were reduced by heating in 2× concentrated reducing sodium dodecyl sulfate–polyacrylamide gel electrophoresis sample buffer (50mM Tris pH 6.8, 2% (w/v) sodium dodecyl sulfate, 10% glycerol, 0.1M dithiothreitol, 5% β-mercaptoethanol, 0.01% bromophenol blue), and equal volumes of proteins were loaded into each lane of a polyacrylamide gel, after appropriate protein measurements. The stacking gel was composed of 4% acrylamide, and the two gradients of the separating gel were composed of 10% and 20% acrylamide, respectively. Prestained color markers (CM; BioRad) and a biotinylated protein ladder for horse radish peroxidase detection (Cell Signalling) were used as molecular mass markers. Separated proteins were blotted overnight onto Hybond-P membrane (polyvinylidenefluoride, GE Healthcare). The blotted polyvinylidenefluoride membrane was then cut into two sections/strips (following CM mass marker), and appropriate parts of the membranes were then immune assayed with their respective antibodies. Membranes for anti-PACAP38 were preblocked with 5%–6% bovine serum albumin and 2% NRS (Normal Rabbit Serum) in Tris-buffered saline containing 0.15% Tween-20 (Tris-buffered saline/T20), and membranes for anti-β-actin were preblocked with 5% milk in 0.15% Tris-buffered saline/T20, for 90 minutes, at room temperature. Primary antibody incubation performed with sheep polyclonal anti-PACAP (1:5,000; Abcam) and mouse monoclonal anti-β-actin (1:4,000; Sigma) overnight at 4°C. Horse radish peroxidase-conjugated rabbit anti-sheep immunoglobin G (1:25,000; Abcam) and goat anti-mouse immunoglobin G (1:1,000; Cell Signalling) were used to detect the bound primary antibodies and were visualized using enhanced chemiluminescence (Immobillon, Millipore). Synthetic PACAP38 was used in every experiment as positive control and run alongside protein samples from young and aged brains, to identify the appropriate *Lymnaea* PACAP38-like bands. Densitometric analysis was performed using ImageJ software from National Institutes of Health. Values for the PACAP38-like bands were normalized relative to their corresponding β-actin bands (used as internal standard). The analysis was repeated 3 times with brain samples obtained from separate batches of animals.

### Statistics

In the behavioral experiments, comparisons between groups were carried out using either *t* tests, nonparametric Mann–Whitney tests, or a one-way analysis of variance, followed by a suitable post hoc test. Parametric tests were only performed on data showing Gaussian distribution. All statistical analyses were carried out using Prism (GraphPad Software). An unpaired *t* test was used to analyze the western blot results. Differences were considered significant at values *p* < .05.

## Results

**Figure 1.**
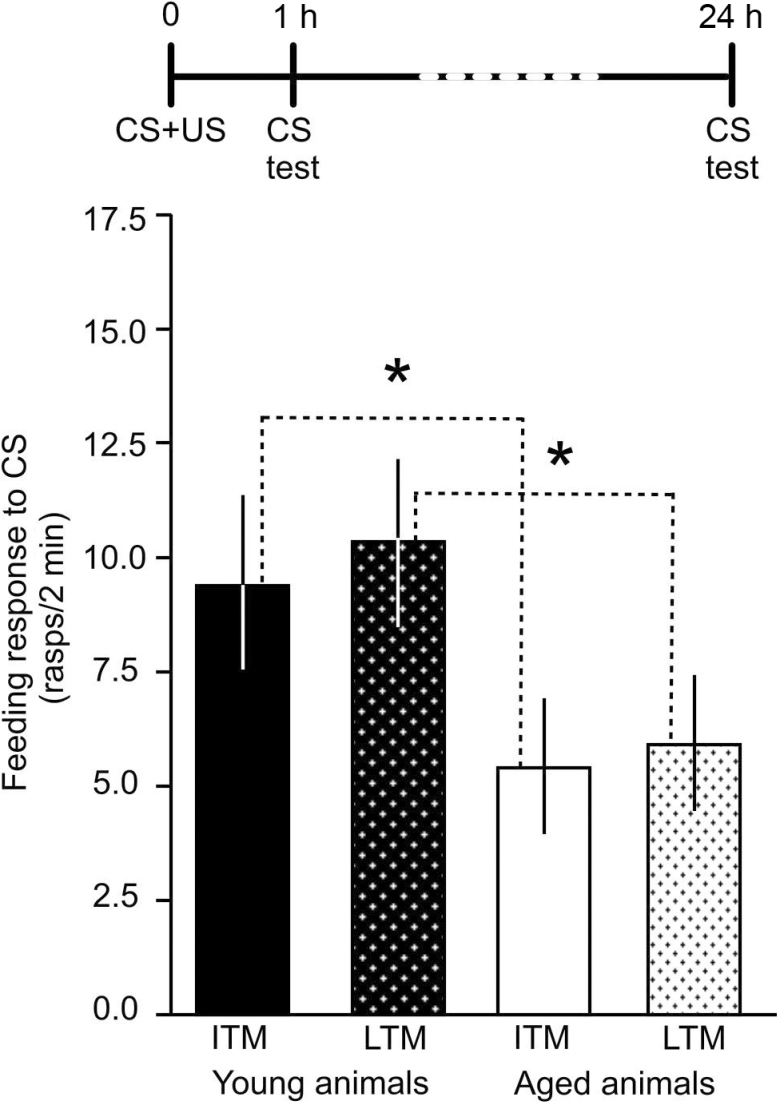
Age-related impairment of both intermediate-term memory (ITM) and long-term memory (LTM) in *Lymnaea*. Time line of experiment is shown above the graph. ITM was tested 1 hour, whereas LTM was tested 24 hours, after single-trial food-reward classical conditioning. Means ± *SEM* are shown. Asterisks indicate statistical significance.

To test the memory retention in young and older snails, a group of 3-month-old (*n* = 18) and another group of 8-month-old snails (*n* = 17) were trained in the single-trial appetitive classical conditioning paradigm. Both groups were tested 1 hour and 24 hours after training. At both testing times, young animals responded significantly higher to the CS than aged ones (Figure 1; Mann–Whitney test, 1 hour: *p* < .0004, 24 hours: *p* < .03), indicating that there is a deficit in the recall of both ITM and LTM in older age. This raised the question whether the reduced ability to recall memory in older age was due to reduced receptor activity.

**Figure 2.**
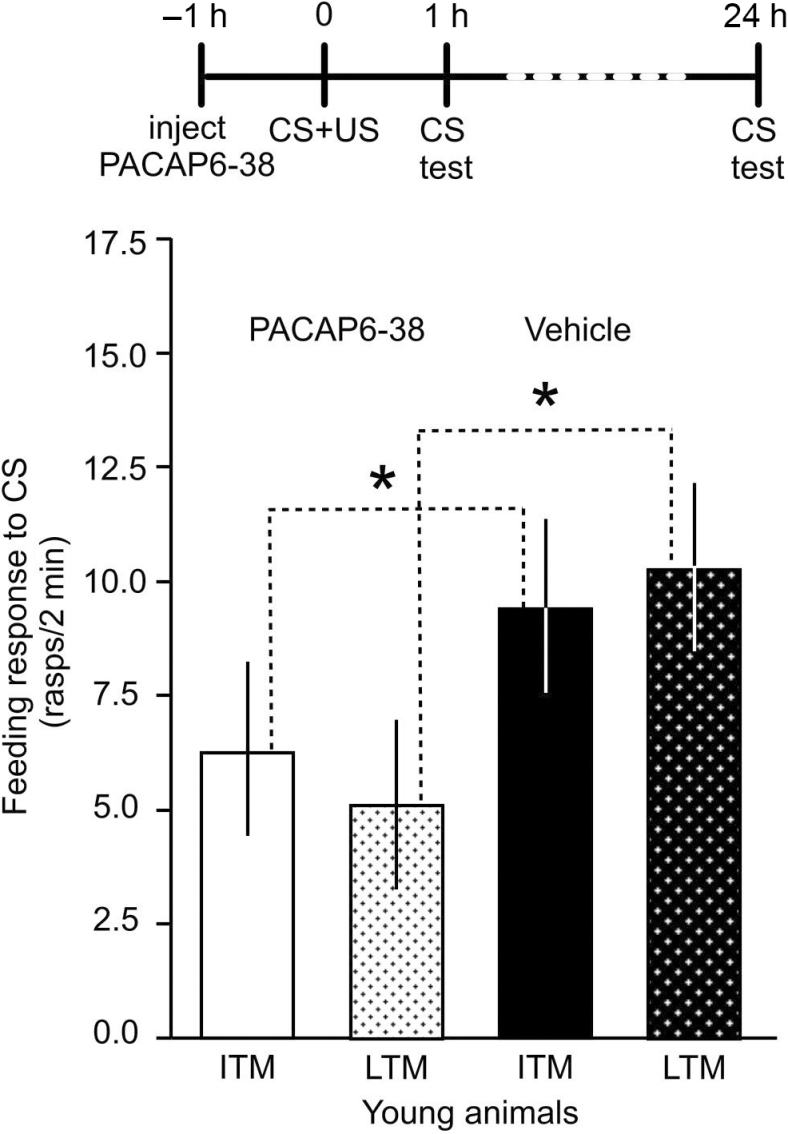
Endogenous pituitary adenylate cyclase activating polypeptide (PACAP) is required for both ITM and LTM in young *Lymnaea*. Time line of experiment is shown above the graph. Injection with the PAC1 antagonist PACAP6-38 (anti-PACAP) 60 minutes before single-trial food-reward classical conditioning caused significant (asterisks) impairment of both ITM and LTM. Means ± *SEM* are shown.

Our previous work (3) showed that the PACAP receptor antagonist PACAP6-38 prevents the formation of LTM in young animals. Here we tested whether ITM formation, 1 hour after training, was also affected. Two groups of young animals were injected either with vehicle solution (*n* = 18) or with PACAP6-38 (*n* = 17). The behavioral tests showed that like the 24-hour memory, the 1-hour memory was also impaired after treatment with the antagonist (Figure 2; Mann–Whitney test, 1 hour: *p* < .001; 24 hours: *p* < .014). These results showed that reduced PACAP receptor activity leads to memory impairment even in young animals both in the intermediate and in the long term.

**Figure 3.**
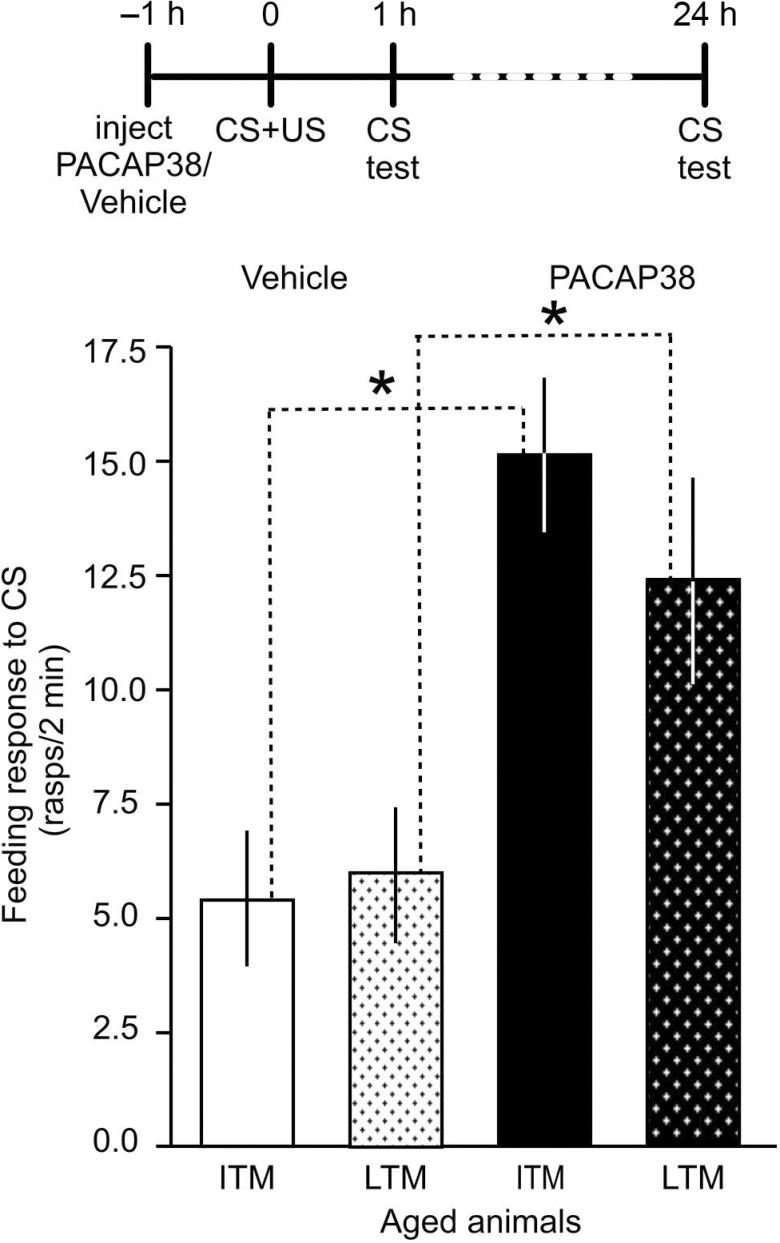
Exogenous PACAP reverses memory impairment in aged *Lymnaea*. Time line of experiment is shown above the graph. Injection with synthetic PACAP38 rescued both ITM and LTM from age-related impairment. Means ± *SEM* are shown. Asterisks indicate statistical significance.

The question then arose whether it is indeed the diminished activity or reduction in the number of PACAP receptors or a reduced PACAP level that causes the defect in memory recall at older age. We hypothesized that if the receptors were affected in older snails, exogenous PACAP could not reverse age-related memory loss. To test this hypothesis, we injected older snails with synthetic PACAP38 to provide plenty of surplus agonist for the receptors during the acquisition and early consolidation of long-term memory. The results of this experiment show (Figure 3) that boosting the availability of PACAP38 during acquisition in older animals could reverse the age-related decline in memory. The comparison of vehicle-injected older animals (*n* = 17) with PACAP38 injected ones (*n* = 16) show that synthetic PACAP38 significantly improved memory at both 1 hour and 24 hours after training (Mann–Whitney test, 1 hour: *p* < .036, 24 hours: *p* < .039). These results indicated that it is the reduced availability of the endogenous PACAP-like polypeptide in aged animals, not the decline in receptors, that leads to memory impairment. To further investigate this possibility, we used western blot techniques to detect differences in the quantity of the *Lymnaea* PACAP-like polypeptide in older and young animals.

**Figure 4.**
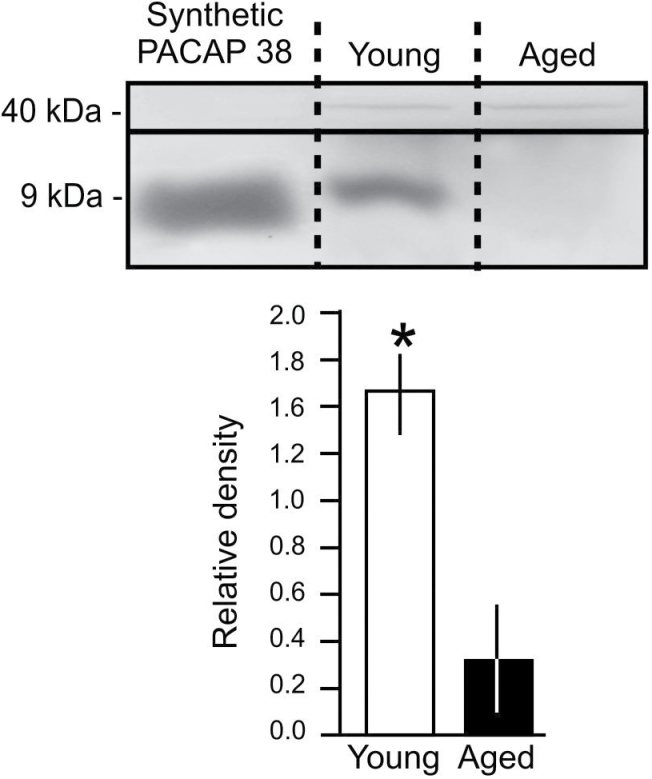
Age-related reduction of endogenous PACAP levels in the “learning ganglia” of *Lymnaea.* (A) Western blot bands of synthetic PACAP38 and endogenous *Lymnaea* PACAP at approximately 9kDa. In both young and aged *Lymnaea*, endogenous actin (~40kDa) was also assayed in the same samples that were used for PACAP detection. The level of endogenous PACAP was below detection threshold in the sample from aged snail brains shown here. (B) Endogenous PACAP expression is significantly (asterisk) lower in aged versus young brain samples. Means ± *SEM* are shown.

An example of the five western blot gels each containing the homogenate of 10 snail buccal and cerebral ganglia (the “learning ganglia” of *Lymnaea* (17)) is shown in Figure 4A. The band visible in the brain sample from young snails is in line with the band of synthetic PACAP38. Densitometry measurements (Figure 4) showed that there was significantly less PACAP38-like peptide in the samples from older snails than in the ones from young animals (two-tailed unpaired *t* test, *p* < .001). This finding confirmed that there was an age-related reduction in the expression of the *Lymnaea* homolog of PACAP in the “learning ganglia.”

**Figure 5.**
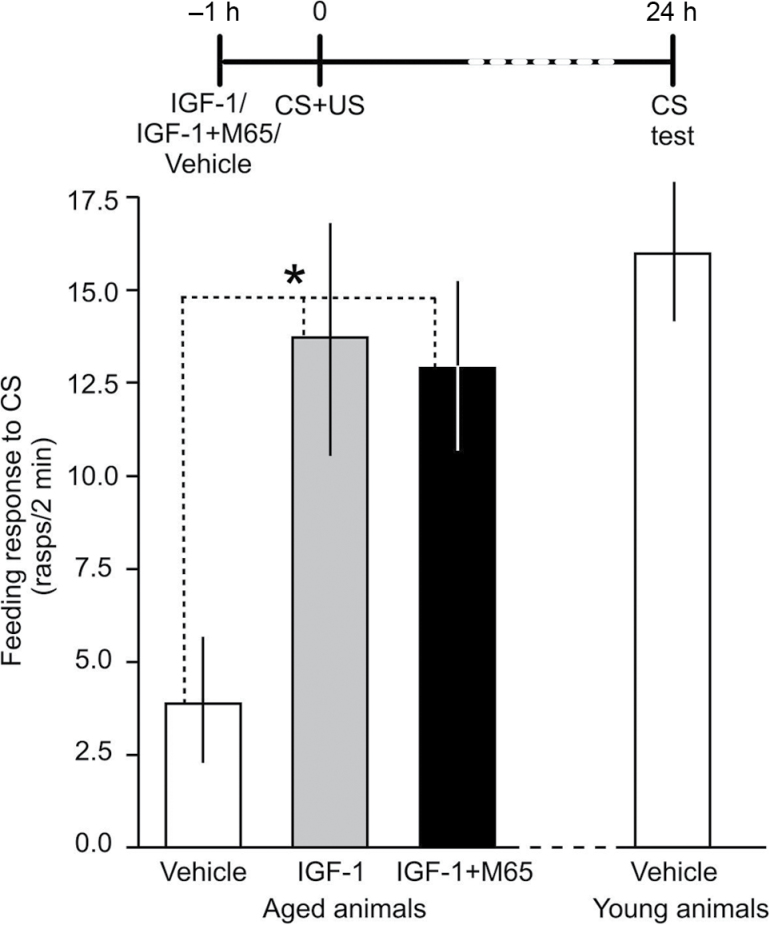
Exogenous insulin-like growth factor-1 reverses memory impairment in aged *Lymnaea*. Time line of experiment is shown above the graph. Similar to PACAP38 (see Figure 3), injection with synthetic insulin-like growth factor-1 rescued LTM from age-related impairment. ITM was not tested in this experiment. For comparison, the conditioned feeding response in vehicle-injected young animals in the same experiment at 24 hours after single-trial classical conditioning is also shown. Means ± *SEM* are shown. Asterisks indicate statistical significance.

There is indication that similarly to PACAP, IGF-1 can also act as a potent neurotrophic and antiapoptotic factor (4). It was believed that IGF-1 exerted its effects via phosphorylation cascades initiated by tyrosine kinase. However, more recent results suggest that it involves a complex signaling network dependent on the PAC1 G-protein-coupled receptor in neurons (4). These findings suggest that IGF-1 could also induce the molecular cascades activated by PACAP through the transactivation of PAC1 receptors. It is also known that IGF-1 plays an important role in mammalian aging (28–37). To test the effect of IGF-1 on associative memory formation in relation to aging, we injected a group of aged snails with recombinant human IGF-1 (R&D Systems, 291-G1) and another group with IGF-1 combined with M65 (PAC1 receptor specific antagonist; Bachem AG, Switzerland, H-6736). The memory test at 24 hours after the training showed that similarly to synthetic PACAP38, IGF-1 boosted long-term memory in older animals (Figure 5). IGF-1 improved memory even when applied together with the PAC1 antagonist (Figure 5), confirming previous findings that IGF-1 transactivates unliganded PAC1 receptors resulting in the phosphorylation state of PKA substrates(4).

## Discussion

Here we introduce a novel invertebrate model of age-related learning deficit and how it can be ameliorated by supplementation with the polypeptides PACAP38 and IGF-1.

A large number of neuropeptides have been linked to both memory function and disease-related dysfunction in mammalian models of learning and memory (38). Although it is known that aging leads to a decline in neuropeptide expression in the brain (39,40), the role of neuropeptide dysregulation in age-associated memory deficits is not well understood. Here, using both a loss-of-function and a gain-of-function approach, we first established a role for a highly conserved polypeptide, PACAP, in age-related memory decline after reward-based classical conditioning.

Previous work (2,15,41,42) already has indicated that PACAP and its most specific receptor (PAC1) exist in the molluscs *Helix* and *Lymnaea*. Pirger and coworkers (3) have also shown that in young *Lymnaea* application of the PACAP receptor antagonist PACAP6-38 around the time of acquisition impairs 24-hour memory (LTM). Our present results confirmed this previous finding and furthermore showed that injecting young (3-month-old) animals with the PACAP receptor antagonist prevented not only LTM but also ITM at 1 hour after training. It seems therefore that in *Lymnaea* PACAP is required for both translation-dependent and transcription-dependent forms of associative memory. In the mammalian brain, the thalamus, hypothalamus, hippocampus (dentate gyrus), cerebellum, and olfactory bulb are the regions containing the PAC1 receptor in the highest density (43). These brain areas are proven to be involved in learning and memory processes and experiments with gene deficient animals (44), and pharmacological interventions (45) indicate a critical role for PACAP in these mechanisms. However, previous work in mammals concentrated on the role of PACAP in learning and memory without studying the involvement of PACAP and its receptors in mechanisms of ITM and LTM during aging.

Our experiments show that in aged *Lymnaea*, there is a significant deficit of both ITM and LTM after one-trial reward conditioning. The deficit in both of these types of memory, however, was rescued by the application of the synthetic PACAP38 peptide before training. The general memory enhancing effect of PACAP has been investigated in mice (12,13) using genetic manipulations and receptor blockers, but no studies were performed with aging animals. We measured the quantity of PACAP in the “learning ganglia” of aged snails and found that it was significantly lower compared with young animals. This finding supports our hypothesis that it is the reduced availability of PACAP that leads to reduction in memory performance in aged animals. Du and coworkers (46) investigated age-related changes in PACAP using similar methods to ours in the gerbil hippocampus at various ages. Their results also indicated that the number of PACAP-immunoreactive cells is lower in the adult stage than in juveniles. However, PACAP immunoreactivity in the mossy fiber zone and PACAP protein level in the hippocampus were highest in the adult stage. This suggests that although there is a global downregulation in the level of PACAP, there are specific regions in the brain where local upregulation can occur. However, establishing the functional consequences of these local differences would need further investigation.

Recently, Lee and coworkers (47) demonstrated that PAC1 receptor immunoreactivity was significantly increased in the hypothalamus, thalamus, midbrain, septal nuclei, and white matter of aged rat brains, whereas VPAC2 receptor immunoreactivity was unchanged. These results also support the notion that it is the level of the available neuropeptide, not the number of receptors, that is responsible for age-related memory deficits.

Importantly, we also found that IGF-1 had a similar memory-boosting effect on aged snails as PACAP38. This can be explained by the previous finding that IGF-1 transactivates PAC1 receptors (4) and thus effectively leads to the activation of the same downstream molecular cascades that are activated by PACAP during memory acquisition.

In a recent study, pharmacological inhibition of phospholipase A_2_ was found to reverse long-term memory impairment and underlying neural defects both in aged and oxidatively stressed young *Lymnaea* (1). This previous study used multitrial operant conditioning of aerial respiration, and it will be interesting to investigate whether phospholipase A_2_ plays a similar key role in age-related memory decline after single-trial classical conditioning. Likewise, it will be interesting to find out if PACAP and IGF-1 have a similar memory rescuing effect on older snails after operant conditioning that we observed here after classical conditioning. The Wildering laboratory used the single-trial food-reward conditioning paradigm to identify the failure of an earlier described type of delayed nonsynaptic neuronal plasticity (48) as an underlying cause of age-related memory impairment (22). It will be therefore important to investigate if PACAP38 or IGF-1 treatment reverses age-related memory impairment by restoring nonsynaptic plasticity in key neurons of the feeding network, similar to the restorative effect of inhibiting phospholipase A_2_ on key neurons of the respiratory network in the case of operant conditioning (1).

Research into age-related behavioral and cellular changes, including memory impairment and underlying neural deficits, have now firmly established *Lymnaea* as a highly tractable model system for exploring the molecular mechanisms of age-associated memory decline. So far phospholipase A_2_ (1), PACAP38, and IGF-1 (this study) have emerged as prospective targets for the reversal of age-related cognitive deficits. These molecules and related biochemical cascades have been highly conserved during evolution and thus the findings from *Lymnaea* can inform new studies exploring these pathways for their role in cognitive decline in the aging human brain and lead to more translationally oriented research aimed at developing new therapeutic approaches to age-associated memory dysfunction.

## Funding

This work was supported by the Hungarian National Scientific Grant (PD109099), "Momentum" research project (LP2011/007), the European Social Fund in the framework of TÁMOP-4.2.4.A/ 2-11/1-2012-0001 "National Excellence Program" (A2-SZGYA-FOK-13-0003), National Brain Research Program (KTIA_NAP_13-2-2014-0006), and Biotechnology and Biological Sciences Research Council grant (BB/KO18515/1).
